# Assessing declarative and procedural knowledge using multiple-choice questions

**DOI:** 10.3402/meo.v18i0.21132

**Published:** 2013-05-22

**Authors:** Ahmed Abu-Zaid, Tehreem A. Khan

**Affiliations:** College of Medicine, Alfaisal University, Riyadh, Saudi Arabia

Assessment is an indispensable aspect of academic life – a natural partner to curriculum and a central component in medical education (1). Aside from its core objective of gauging student performance, assessment is also crucial for documenting the attainment of learning outcomes, verifying medical competence and, ultimately, minimizing risks to patients arising from an unsatisfactory knowledge base (2).

For ‘traditional’ written exams, it is crucially important to generate high-quality multiple-choice questions (MCQs) for formative and summative assessments (3).

In both applications, MCQs should promote effective learning that, in turn, prepares trainees for safe and competent clinical practice. As such, there is an increasing need for MCQs that examine declarative and procedural knowledge, since both are essential in assessing knowledge from a different perspective.

Declarative knowledge MCQs are defined as questions that assess ‘pure recall’ of specific isolated pieces of knowledge such as facts, definitions, terminologies, concepts, etc. (4). An example of a declarative knowledge MCQ will be: which organ of the body produces insulin? On the contrary, procedural knowledge MCQs are defined as questions that assess ‘problem-solving skills’ in understanding concepts, assembling knowledge from across varying scientific disciplines, making rational predictions, applying critical judgments, arriving at conclusions, and deciding on the best course of actions (4). An example of a procedural knowledge MCQ will be: a 55-year-old patient who presented with high-grade fever, significant weight loss of 15%, excessive night sweats, lower abdominal pain and massive volume of blood in stools. What is the best next step in the management of this patient?

To properly process procedural knowledge in reaching conclusions or to make wise clinical judgments, medical students must have a solid foundation of declarative knowledge upon which to build (4). However, in achieving this, educators must minimize the ‘blunt’ short-term memorization of declarative knowledge, which neither results in actual learning nor clinically-useful application. In summary, MCQs tapping declarative knowledge should be given equal credence (with procedural knowledge MCQs) in curricular assessments.

Reflecting current trends, formal assessments of procedural knowledge are important in honing problem-solving and critical-thinking skills (5), as well as nurturing the development of life-long learning. However, such procedural knowledge should itself be assessed on more than mere short-term knowledge retention – focusing instead on the comprehension and long-term retrieval of scientific knowledge readily applicable to a range of problem-based, real-life clinical situations. Moreover, MCQs measuring procedural knowledge should encourage the use of higher-level cognition geared toward critical appraisal and problem-solving skills.

With regards to MCQs targeting procedural knowledge, it is important to construct questions that explore a deeper understanding of basic science content which demand the use of higher-order thinking skills to integrate such knowledge with other scientific principles in vis-a-vis relevant, clinically-oriented contexts. These practices, in turn, promote longer-term retention of knowledge and prepare medical students for competent patient care.

While crafting declarative knowledge MCQs is fairly simple, structuring procedural knowledge MCQs can be challenging (6). Using short-answer questions (SAQs) requiring problem-solving is one method that allows for the integration of basic and clinical science (7) – and can explore students’ knowledge application, problem-solving skills, clinical reasoning abilities, critical appraisal capacities and management proficiencies in the context of clinically-based cases. Of course, as with the measurement of any construct, the use of multiple assessment methods is advisable (1).

For educators, assessment is not simply a tool for measuring knowledge gain. Rather, it is also a process that can significantly guide and organize students’ learning (8) into surface (memorizing facts), deep (understanding concepts) or strategic (assessment-driven) approaches (9). To exemplify, declarative knowledge MCQs typically promote surface learning, whereas procedural knowledge MCQs are capable of promoting deep learning. Obviously, in medical training, the latter is most desired as it allows for the application of theoretical knowledge to clinical practice and, hence, promotes the learning of procedural knowledge.

MCQs, if properly formatted, are a valuable means of guiding students toward the desired learning outcomes, and can help to ensure competent physicians who can integrate scientifically-correct (declarative) with scientifically-sound (procedural) knowledge.
